# VARIABLES INVOLVED IN THE MANAGEMENT OF SCHOOL BULLYING: A BAYESIAN NETWORK ANALYSIS

**DOI:** 10.1590/1984-0462/2021/39/2019079

**Published:** 2020-08-26

**Authors:** Mariá Romanio Bitencourt, Tatiana Sayuri Hizukuri, Marcos Rogério Bitencourt, Ana Carolina Jacinto Alarcão, Elias César Araújo de Carvalho, Luciano de Andrade, Sandra Marisa Pelloso, Maria Dalva de Barros Carvalho

**Affiliations:** a Universidade Estadual de Maringá, Maringá, PR, Brazil.

**Keywords:** Bullying, Schools, Violence, Children, Bullying, Escola, Violência, Criança

## Abstract

**Objective:**

To analyze the management of bullying by the managers of elementary schools.

**Methods:**

Descriptive, exploratory research carried out through semi-structured interviews with 17 school counselors from a city in the South of Brazil, randomly selected from different geographical sectors. The interviews were recorded with participants’ consent and, after transcription and checking, were discarded. The interviews covered the following subjects: sociodemographic characterization of subjects, school functioning, comprehension, recognition and management of bullying cases by counselors. Data analysis was performed using the Bayesian network associated with content analysis.

**Results:**

The majority of subjects were females, between 30 and 50 years old. Fifteen subjects were graduated in pedagogy, and all had postgraduate degrees. Most of them worked as counselor for less than three years. Only two subjects, between 30 and 50 years old, understood the term *bullying.* Case recognition was lower in this age group. Having a degree influenced positively the recognition of bullying. The higher the number of students in the school, the lower the recognition of cases by managers. All subjects managed cases by addressing children, families, staff, and involving professionals and support groups.

**Conclusions:**

The understanding and recognition of bullying was given by a few interviewees. All managers reported similar management actions in the cases. Given the scarcity of studies on bullying management in schools, more studies in this area could improve the approach of cases and contribute to their reduction.

## RESUMO

**Objetivo:**

Analisar o gerenciamento de conflitos do tipo *bullying* pelos gestores de escolas de ensino fundamental.

**Métodos:**

Pesquisa descritiva e exploratória realizada por meio de entrevistas semiestruturadas com 17 orientadores de escolas escolhidas aleatoriamente de um município do Sul do Brasil, contemplando os diversos setores geográficos locais. As entrevistas foram gravadas mediante consentimento, e, após transcrição e conferência pelos sujeitos, descartadas. As entrevistas abordam: caracterização sociodemográfica dos sujeitos, funcionamento da escola e compreensão, reconhecimento e gerenciamento dos casos de *bullying* pelos orientadores*.* A análise dos dados foi feita com base na rede bayesiana associada à análise de conteúdo.

**Resultados:**

A maioria dos sujeitos era do sexo feminino e tinha entre 30 e 50 anos. Quinze sujeitos eram formados em pedagogia, e todos possuíam pós-graduação. A maioria atuava na função de orientador havia menos de três anos. A compreensão do termo *bullying* deu-se apenas por dois sujeitos, com idade entre 30 e 50 anos. Já o reconhecimento dos casos foi menor nessa faixa etária. Ter feito pós-graduação influenciou positivamente o reconhecimento de *bullying*. Quanto maior o número de alunos na escola, menor o reconhecimento dos casos pelos gestores*.* Todos os sujeitos gerenciaram os casos abordando as crianças, famílias e equipe e envolvendo profissionais e núcleos de apoio.

**Conclusões:**

A compreensão e o reconhecimento do *bullying* deram-se por poucos entrevistados. Todos os gestores relataram ações de gerenciamento semelhantes diante dos casos. Tendo em vista a escassez de estudos sobre gestão de *bullying* na escola, mais estudos nessa área poderiam melhorar a abordagem dos casos, contribuindo para sua redução.

## INTRODUCTION

The increase in prevalence of bullying episodes in different cultures and its consequences for the ones involved has turned it into a public health problem.^1^ ^,^ ^2^ According to data from the United Nations (UN), in Brazil, the rate of occurrence of bullying among children and young people in 2016 was 43%. Developed countries also have high percentages: Germany (35.7%), Norway (40.4%) and Spain (39.8%).^3^ Bullying is the most common form of violence in the school environment.^4^ In the United States, the prevalence is 20% among high school students;^5^ and in Ireland, 11.8% among students aged 12 to 18 years and 22.4% among students aged four to 13 years.^6^

The school climate, the teaching-learning process, as well as the health and development of children and adolescents are affected by bullying,^1^ ^,^ ^2^ which can cause physical, psychological, social or educational damages^7^ that affect victims, bullies and observers.^8^ Damages resulting from this type of violence are a worrying reality in our schools due to the immediate and future consequences that they can cause.^9^ Therefore, school professionals must be familiar with the problem so that they can approach bullies, victims and observers, in addition to families and school staff. Proper management of these cases by educators is an important factor to reduce bullying in schools.^10^

The literature brings studies on bullying that address teachers’ knowledge on the topic,^11^ the occurrence of suicide among victims,^12^ cyberbullying^13^ and bullying observers.^14^ As far as it is known, there are no studies covering school managers or the variables involved in the identification and management of bullying cases from first to fifth grade of elementary school.

The aim of this study was to analyze the predictive factors for bullying and the variables involved in the knowledge, recognition and management of these conflicts by school managers in a municipality in southern Brazil.

## METHOD

This is a descriptive and exploratory research conducted with managers (supervisors) of municipal elementary schools from the first to the fifth grade of a medium-sized and planned city, with an area of 487,930 km^2^, in the State of Paraná. According to data from the Brazilian Institute of Geography and Statistics (IBGE), Pará has 417,010 inhabitants, approximately 45,000 children aged 5 to 14 years old, and its human development index (HDI) is 0.808.

The 55 municipal schools were mapped using geographic coordinates, using the geographic mapping software QGIS version 2.8.3,^15^, which randomly selected 20 schools representing different regions of the city’s urban perimeter: central, intermediate and peripheral.

A pilot project was carried out to test the questionnaire, aiming at verifying whether the data collection instrument offered conditions to achieve the objectives proposed. Semi-structured interviews were carried out with the principals, supervisors and counselors of two schools, so the closest manager who is familiar with bullying conflicts could be chosen. The counselor was the most capable professional to answer the research questionings. Interviews with other professionals were excluded from the study.

Data collection took place from November to December 2017, and the interviews were made at the schools, according to participants’ availability, and lasted about 30 minutes. Fourteen schools participated, considering the data saturation criterion, totaling 17 subjects. Three schools had two counselors. It is worth mentioning that there was no refusal on the part of the other six schools. The criterion used was data saturation. That is, the answers began to be repeated from the tenth school surveyed. Even so, we advanced to the 14th school, to check if new data would appear, which did not happen. Thus, we decided to end data collection.

The interview addressed personal information of the subjects (name, sex, age, time of management at school, training, religion) and questions about the functioning of the school (total number of students per classroom, number of full-time students, functioning of start and end of school shift, and breaks). The third part addressed bullying (subject’s understanding of the term and experience at the school, how the subject recognizes cases, what is the profile of a child that is most susceptible to being bullied and what signs they show, what places are most prone to the occurrence, what are the consequences for the victim, and how the issue is handled with the parts involved).

The data from the interview were recorded with participants’ consent and fully transcribed. After checking and validation of texts by the subjects, in person or via e-mail, the recordings were disposed of. The subjects could agree with or disagree with the answers and allowed to correct them.

Data analysis was carried out through content analysis in the thematic mode, based on Minayo’s recommendations.^16^ In order to increase the understanding of the relationships between the variables involved in bullying management, several Bayesian networks (BN) were built using the package R Bayesian Networks & Path Analysis (BNPA).^17^ The non-parametric bootstrap method^18^ was used to estimate the accuracy of BNs.

After the creation of the BNs, a group of two researchers (MDBC, ACJA), PhDs, with a background in research on bullying, separately evaluated each BN structure generated. As a concept used to choose the BN structure that could best represent the causal relationship of this study, we considered:

- The largest number of possible predictors for the outcome variable presented in the literature.
- The smallest number of incorrect relations.
- The smallest number of isolated variables (without relations) or meaningless subgraphs.

After individual evaluation of each researcher, both met to reach a consensus on possible discrepancies. In case there was no consensus, a third researcher (SMP) would give an opinion. With the chosen BN structure, a polychoric correlation analysis was performed in the software R polycor,^19^ to determine whether the relation between the variables was positive or negative.

The original database was composed of 14 variables and 17 records. The variable names were converted. Table 1 contains the original label, the new label and the caption of the variables. The data set had no missing data. The multicollinearity check identified independent variables with more than 0.90 correlation with other independent variables: full-time (FT), break with all age ranges together (ART), entry and exit common to all age groups (EET), religion (REL), sex (SEX) and management of bullying (MBY). These variables were removed, totaling eight: age (AGE), management time at school (MTS), training in pedagogy (TP), knowledge about bullying (KBY), postgraduate degree (PGD), total number of students at school (NSS), number of students per class (NSC) and recognition of bullying (RBY).

This study was approved by the Permanent Research Ethics Committee of Universidade Estadual de Maringá (Opinion nº 2,230,881).

**Table 1:** Data set.

Original label	New label	Description
Management time at school	MTS	Time as a manager at school (in years) (< or >3 years)
Training in pedagogy	TP	Having a degree/training in pedagogy
Postgraduation in management	PGD	Having a postgraduate degree in school management
Number of students at school	NSS	Number of students at school (< or >500 students)
Number of students per class	NSC	Number of students per class (< or >30 students)
Age	AGE	30-50 years or >50 years
Full time	FT	Full-time shift
Common break	CB	Break with all age groups at once
Common entry-exit time	EET	Entry and exit of all students at the same place and time
Religion	REL	Catholic or Protestant
Gender	SEX	Female/male
Knowledge about bullying	KBY	Yes or no
Recognition of bullying	RBY	Yes or no
Management of bullying	MBY	Yes or no

## RESULTS

Seventeen mentors aged between 37 and 63 years participated in the study. They were divided into two categories, 30 to 50 years old (52.9%) and over 50 years old. Sixteen of them (94.1%) were females. The Catholic religion was predominant among participants (52.9%). Fifteen subjects (88.2%) had a degree in pedagogy, and most had been in school orientation for less than three years. All respondents had postgraduate degrees: neuropedagogy, special education and school management were the most common areas mentioned.

In 85.7% of schools, between 100 and 500 students are enrolled. Eleven schools (78.5%) offer full-time shift. All schools have 20 to 35 students per class. The entry and exit times are the same for different age groups in 12 schools (85.7%). The breaks are separated by age groups in 13 schools (92.8%), that is, the first and second years in one environment and the third, fourth and fifth years in another.

After exhaustive reading of the managers’ answers, three inductive categories were outlined: bullying understanding/knowledge, recognition and management.

### Understanding of bullying

Based on the concepts and ideas seen in answers, two dichotomous subcategories were created:

- Understands bullying: answers including concepts such as violence, repetition and intentional character.
- Does not understand bullying: incomplete answers involving child conflicts, wide dissemination of the topic and trivialization of the term.

### Recognition of cases of bullying

The recognition of bullying cases by managers was based on the observation of signs and symptoms of children who suffer it, the profiles of the most affected ones and the places of frequent occurrence. The terms “obese” and “skin color” were cited as the profiles of children most susceptible to becoming victims of bullying. The most propitious place or time for the occurrence was the break time. Isolation, sadness and low self-esteem were the signs seen in the victims, being also mentioned: change of mood, irritation, crying, lack of attention, school absences, frequent pain complaints, fear of going to school, and drop in school performance.

To assist in the understanding and recognition of bullying, the BN was used. Tables 2 and 3 show the results obtained after the bootstrap process during the creation of the BN structure. The “From” column represents the source variable, “To” points to the target variable, “Power” indicates the probability that there is an arc between these variables, and “Direction” is a parameter that must contain values above 0.50, which suggests a support to that relationship direction.

**Table 2:** Result of the bootstrap process for understanding of bullying.

From	To	Power	Direction
MTS	KBY	0.66	1.00
MTS	TP	0.71	0.76
PGD	KBY	0.83	1.00
AGE	KBY	0.85	1.00
AGE	TP	0.86	1.00
AGE	MTS	0.95	1.00

MTS: management time at school; PGD: postgraduate degree; AGE: age; KBY: knowledge about bullying; TP: training in pedagogy

**Table 3:** Result of the bootstrap process for recognition of bullying.

From	To	Power	Direction
MTS	TP	0.68	0.73
NSS	RBY	0.76	1.00
PGD	RBY	0.79	1.00
NSC	RBY	0.93	1.00
AGE	MTS	0.94	1.00
AGE	RBY	0.94	1.00
MTS	RBY	0.94	1.00

MTS: management time at school; NSS: number of students at school; PGD: postgraduate degree; NSC: number of students per class; AGE: age; TP: training in pedagogy; RBY: recognition of bullying

The choice of the most adequate BN structure considered the graph with the largest number of predictors possible and the lowest number of incorrect relationships. The category “management of cases” did not allow the use of the BN method, as all subjects reported management actions, with no sufficient variance to perform statistical or probability calculations required by the methodology.

**Figure 1:**
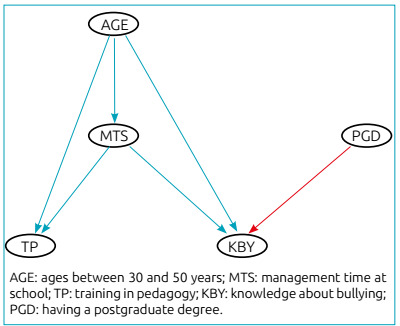
Bayesian network representing the causal relationship for the understanding of bullying.

The graphic representation of positive or negative influence was given by the colors blue and red, respectively. The thickness of the rod is related to the intensity of the influence. Figure 1 represents the BN created for the causal relationship between predictor variables of the outcome variable “knowledge about bullying”. In this case, the age variable had a ­positive influence, as well as MTS. The PGD variable had a negative ­influence on KBY.

**Figure 2:**
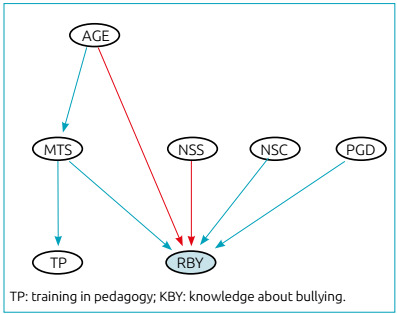
Bayesian network created for the causal relationship between the predictive variables of recognition of bullying (RBY). The variables management time at school (MTS), number of students per class (NSC) and having a postgraduate degree (PGD) were identified as a positive influence; the variables age between 30 and 50 years (AGE) and number of students at school (NSS) were considered negative influence on the KBY variable.

Figure 2 represents the BN created for the causal relationship between predictive variables of RBY. The variables MTS, NSS and PGD were identified as a positive influence on the recognition of the variable age, and NSC, as a negative influence on the variable RBY.

### Management of bullying conflicts

The management of this type of conflict in the school environment was subcategorized through the analysis of the answers related to:

- Approaching parts involved: victims, bullies, observers, support professionals, families.
- Multiprofessional discussion: with professionals from the Basic Health Unit (UBS) and from the Municipal Center for Specialized Support (CEMAE), psychologists.

## DISCUSSION

To date and to our knowledge, this study is the first to analyze the management by school administrators of bullying conflicts in schools with students aged 6 to 12 years. It is also the first to use BN structure learning algorithms to assess the causal influence of possible predictors for knowledge about and recognition of cases of bullying.

Regarding the category “knowledge about bullying”, the subjects cited the concepts that the literature identifies as bullying: violence, intention to cause harm and repetition - findings corroborated by Olweus.^20^ This may have occurred due to the understanding of the term being more related to intrinsic values of the subject than with pedagogical or postgraduate training in the area of school management, or even the time of acting as a manager. Lima et al.,^21^ in a study conducted with teachers, they demonstrated to know the term bullying, but did not feel prepared to manage the problem and revealed the need for training. In the present study, the subjects reported having access to trainings and did not express management difficulties.

Bullying was described as a phenomenon that has always existed, but currently, due to the greater importance given to the theme, is trivialized. Managers in the age group 30 to 50 years showed a better understanding of the cases, perhaps due to their familiarity with the concept. Subjects over 50 years old, on the other hand, may have experienced similar conflicts in the past, but these were not called bullying back then or were given the importance they are given today, making understanding difficult.

The length of experience of less than three years in school management had a positive influence on the understanding of bullying, and it is worth highlighting the fact that these managers have a lot of experience in teaching. In the study by Silva and Rosa,^11^ only one teacher among the six interviewees stated that the topic of bullying was addressed during their teacher training. As the participants in that study already had more than 20 years of teaching, the authors believe that during their initial training, the discussion about bullying was not part of the content repertoire.

Having a background in pedagogy or a postgraduate degree in school management did not influence in understanding of bullying. These data are in agreement with the study by Bandeira and Hutz,^22^ which identified that the lack of knowledge about the way bullying presents itself and spreads can contribute to omission because of professional unpreparedness and lack of knowledge on how to solve the problem. The importance of a reformulation in teacher education is also highlighted, as it needs a greater focus on violence at school. The lack of information about the characteristics of school bullying by teachers can lead to difficulties in recognizing the context of victimization and aggression. Lima et al.^21^ believe that this stems from failures in continuing education regarding school violence. In the present study, the subjects mentioned participating in trainings frequently.

Professionals believe that there is more bullying in other schools than in the schools in which they operate. This can result from the wide dissemination of the topic in the media, the lack of information about the cases in their own school, or the difficulty or fear of reporting the cases.^23^ In the 2012 National School Health Survey (PeNSE) carried out with ninth-year students, 7.2% of them reported suffering bullying and 20.5% confessed practicing it.^24^ The percentage of cases is not low, even though we have approached a younger age group. Managers who believe that there are no cases of bullying in their schools may not understand or recognize such cases, which makes it difficult to manage.

Regarding the variables involved in the profile of susceptibility to bullying, obesity was the most cited characteristic. The same was reported by Juvonen and Graham,^23^ who highlighted the issue as an increased risk of being bullied.

Another variable involved in bullying was skin color. PeNSE 2012^24^ reported that black-skin students were the ones who most claimed to not be treated well in the last month and were also the ones who most practiced bullying.

Some signs and symptoms presented by the victims and reported by the managers are in accordance with Silva and Costa’ study,^25^ in which change of mood, irritation, crying, lack of attention, school absences and frequent pain stood out. The same applied to fear of going to school, drop in school performance and low self-esteem, also pointed out by Kim and Kim^26^ in their review article.

In addition to the victims, the bullies show signs in line with those found by Oliveira et al.:^27^ they feel lonely, they have no friends, they have more school absences and they suffer more family violence. These data reinforce the importance of approaching not only the victim, but also the bully and the observers, who also suffer the consequences of this type of violence.

Self-esteem is a factor of emotional protection and well-being when facing difficulties in childhood and pre-adolescence.^28^ In our study, low self-esteem was mentioned as a consequence presented by children who are victims of bullying, which reinforces the need for management and prevention of these events, considering the formation of children’s personality.

For Silva and Costa,^25^ in Brazil, bullying occurs more frequently in the classroom, in contrast to international surveys, which indicate a higher frequency in breaks and times of entry and exit. In the present study, the break time was cited as the main moment, as there is less supervision by school authorities, as shown by the study by Majcherová et al.^29^

Another variable involved was the issue of managing bullying cases. Such management through the approach in the classroom was mentioned by the research subjects and also in the study by Silva and Costa,^25^ in which works were developed in the classroom with the use of texts, comic books, videos and lectures on the topic.

The best way to reduce bullying after the fact occurred is through conversations with the family.^25^ In our study, managers said they used the strategy as a way to manage and reduce conflicts.

Another way of managing bullying cases is to involve other professionals, for example, requesting the presence of a social worker in the school premises, since they have different contacts with government agencies and associations.^29^ In the studied municipality, social workers are present at the Social Assistance Reference Center (CRAS) and in basic health units, which are services that can assist in the management and prevention of cases.

For Silva and Costa,^25^ there must be partnerships between schools, families and sectors of society in order to reduce violence. In addition, anti-bullying campaigns must be promoted to improve case identification. The authors also suggest the presence of the psychopedagogue to develop a work with children, families and schools, making them aware of the importance of their conduct. In the present study, the psychopedagogists at CEMAE were referred by the subjects as professionals who they rely on in these cases.

No specific referrals to the pediatrician were mentioned; however, as this is a health issue, this professional could assist in the diagnosis as part of a multidisciplinary approach, as proposed by Kim and Kim.^26^

This study had some limitations. One of them was the involvement of only public-school managers. To lighten this limitation, schools from different regions of the municipality were evaluated. It was noted that respondents had difficulty in acknowledging bullying in the schools where they work. In order to resolve this limitation, in addition to discourse analysis, BN were used. Finally, another limitation was the study being carried out in a medium-size municipality. However, the results found tend to be common to other populations in the same circumstances.

We conclude that bullying is still a topic that presents difficulties in understanding, recognition and management by school professionals. The approach should be made by a multidisciplinary team, involving teachers, counselors, psychologists, pediatricians, social workers and support bodies working with families.

The prevention of school violence is a demanding task that requires the involvement of managers in the fields of education and health, to avoid further consequences, thus making the school a more pleasant and safe environment.

## References

[1] Jones SN, Waite R, Clements PT (2012). An evolutionary concept analysis of school violence: from
bullying to death. J Forensic Nurs.

[2] Rech RR, Halpern R, Tedesco A, Santos DF (2013). Prevalence and characteristics of victims and perpetrators of
bullying. J Pediatr (Rio J).

[3] Organização das Nações Unidas (2016). Pesquisa da ONU mostra que metade das crianças e jovens do mundo já
sofreu bullying.

[4] Menesini E, Salmivalli C (2017). Bullying in schools: the state of knowledge and effective
interventions. Psychol Health Med.

[5] Eaton DK, Kann L, Kinchen S, Shanklin S, Flint KH, Hawkins J (2012). Youth risk behavior surveillance - United States,
2011. MMWR Surveill Summ.

[6] Foody M, Murphy H, Downers P, Norman JO (2018). Anti-bullying procedures for schools in Ireland: principals’
responses and perceptions. Pastor Care Educ.

[7] Gladden RM, Vivolo-Kantor AM, Hamburger ME, Lumpkin CD (2014). Bullying surveillance among youths: uniform definitions for public
health and recommended data elements.

[8] Wolke D, Lereya ST (2015). Long-term effects of bullying. Arch Dis Child.

[9] Silva D, Tavares E, Silva ES, Duarte J, Cabral L, Martins C (2017). Victims and aggressors - bullying manifestations in students from
6th to the 9th grade schooling. Rev Port Enferm Saude Mental.

[10] Burger C, Strohmeier D, Sproeber N, Bauman S, Rigby K (2015). How teachers respond to school bullying: an examination of
self-reported intervention strategy use, moderator effects, and concurrent
use of multiple strategies. Teach Teach Educ.

[11] Silva EM, Rosa EC (2013). Do teachers know what is bullying? A teacher training
issue. Psicol Escolar Educ.

[12] Klomek AB, Snir A, Apter A, Carli V, Wasserman C, Hadlaczky G (2016). Association between victimization by bullying and direct
self-injurious behavior among adolescence in Europe: a ten-country
study. Eur Child Adolesc Psychiatry.

[13] Modecki KL, Minchin J, Harbaugh AG, Guerra NG, Runions KC (2014). Bullying prevalence across contexts: a meta-analysis measuring
cyber and traditional bullying. J Adolesc Health.

[14] Rivers I, Noret N (2013). Potential suicide ideation and its association with observing
bullying at school. J Adolesc Health.

[15] Sutton T Documentation for QGIS 1.8.

[16] Minayo MC (2006). O desafio do conhecimento: pesquisa qualitativa em saúde.

[17] Carvalho E, Vissoci JR, Andrade L, Cabrera EP, Nievola JC (2018). BNPA: Bayesian networks & path analysis. R package version
0.3.2..

[18] Friedman N, Halpern JY (1999). Data analysis with Bayesian networks: a bootstrap approach.

[19] Fox J (2016). Polycor: polychoric and polyserial correlations. R package version
0.7-5.

[20] Olweus D (2013). School bullying: development and some important
challenges. Annu Rev Clin Psychol.

[21] Lima RF, Jager ML, Souto DC, Martins CA, Dias AC (2013). The perception of teachers on school bullying. Discip Sci Sér Ciênc Biol Saúde.

[22] Bandeira CD, Hutz CS (2012). Bullying: prevalence, implications and gender
differences. Psicol Esc Educ.

[23] Juvonen J, Graham S (2014). Bullying in schools: the power of bullies and the plight of
victims. Annu Rev Psychol.

[24] Malta DC, Prado RR, Dias AJ, Mello FC, Silva AI, Costa MR (2014). Bullying and associated factors among Brazilian adolescents:
analysis of the National Adolescent School-based Health Survey (PeNSE
2012). Rev Bras Epidemiol.

[25] Silva AC, Costa AM (2014). The role of psychopedagogists relative to
bullying. Rev Psicopedag.

[26] Kim SK, Kim NS (2013). The role of the pediatrician in youth violence
prevention. Korean J Pediatr.

[27] Oliveira WA, Silva MA, Silva JL, Mello FC, Prado RR, Malta DC (2016). Associations between the practice of bullying and individual and
contextual variables from the aggressors’ perspective. J Pediatr (Rio J).

[28] Tambelli R, Lagh F, Odorisio F, Notari V (2012). Attachment relationships and internalizing and externalizing
problems among Italian adolescents. Child Youth Serv Rev.

[29] Majcherová K, Hajduová Z, Andrejkovič M (2014). The role of the school in handling the problem of
bullying. Aggress Violent Behav.

